# Stage-Specific Healthcare Costs in Cervical Cancer and Cervical Intraepithelial Neoplasia: A Population-Based Analysis Informing Value-Based Oncology and Equitable Prevention

**DOI:** 10.3390/curroncol33060329

**Published:** 2026-06-01

**Authors:** Tian-Shyug Lee, Yu-Chiao Wang

**Affiliations:** 1 Graduate Institute of Business Administration, College of Management, Fu Jen Catholic University, New Taipei City 242062, Taiwan; 036665@mail.fju.edu.tw; 2 Department of Healthcare Management, Yuanpei University of Medical Technology, Hsinchu 300102, Taiwan

**Keywords:** cervical intraepithelial neoplasia, invasive cervical cancer, direct medical costs, Taiwan

## Abstract

Persistent challenges in cervical cancer (CC) control highlight the need for stage-specific cost estimates to refine prevention strategies. Structural integration of National Health Insurance (NHI) administrative claims, the Taiwan Cancer Registry (TCR), and the National Cause of Death Registry (NCDR) provided the empirical basis for this population-based research. The final analytical sample encompassed 6055 women with cervical intraepithelial neoplasia (CIN) identified in 2016 as well as 9318 patients diagnosed with stage I to IV invasive CC during the 2008 to 2015 period. Reimbursed direct medical costs were estimated for CIN within 6 months after diagnosis and for CC over 5 years after diagnosis. Across CIN grades, no consistent cost gradient was observed, although inpatient utilization was highest in CIN3. Among women with CC, healthcare utilization and expenditures were concentrated in the first year after diagnosis, accounting for 52–65% of the total 5-year costs. After age adjustment, the mean first-year costs increased from NT$256,095 (US$8413) in stage I to NT$474,724 (US$15,595) in stage IV, while 5-year survival declined from 85.3% to 19.5%. These findings show that cervical disease imposes substantial direct medical costs on Taiwan’s healthcare system and provide updated evidence to inform human papillomavirus (HPV) vaccination and CC screening policy.

## Simple Summary

The clinical manageability of cervical cancer (CC) does not negate the persistent economic strain it imposes on public health systems. Leveraging nationwide Taiwanese datasets, this research mapped medical expenditures across the transition from precancerous lesions to malignancy. Rising clinical severity dictates the trajectory of medical spending. Data show that the heaviest fiscal burden occurs during the first twelve months after diagnosis. The resource intensity in oncology typically escalates alongside disease progression. Advanced malignancies require substantial medical spending for limited survival gains, whereas early-stage interventions offer greater clinical value. This disparity provides a clear economic rationale for prioritizing early detection. These cost-based findings offer an empirical basis for refining national screening and vaccination policies to balance patient outcomes with health system sustainability.

## 1. Introduction

Cervical cancer (CC) is one of the few cancers with a clearly established direct cause. Clinical evidence attributes almost all invasive cases to persistent high-risk human papillomavirus (HPV) infection [1,2]. In immunocompetent women, the World Health Organization (WHO) notes that malignant transformation from early cellular changes to invasive disease generally takes 15 to 20 years. This latency period, however, shortens considerably when immune function is compromised. Disease progression is also influenced by other recognized risk factors, including human immunodeficiency virus (HIV) co-infection, smoking, multiparity, and prolonged use of hormonal contraceptives [2]. This naturally extended precancerous phase provides a crucial window for clinical prevention and intervention. By combining HPV vaccination, routine screening protocols, and the early treatment of precursor lesions, the development of CC can be effectively prevented [2,3,4].

Long regarded strictly as a preventable condition, CC is currently the subject of a worldwide elimination campaign. The WHO sets a strict quantitative threshold for this elimination: an age-standardized incidence of fewer than 4 cases per 100,000 women. The core strategy to reach this figure is the “90–70–90” framework. These benchmarks require broad HPV vaccination, screening with a high-performance test at ages 35 and 45, and timely treatment for detected precancerous lesions or invasive tumors [3]. To align with these global goals, the standard of care has transitioned toward more effective diagnostic methods. The second edition of the WHO guidelines specifically recommends HPV DNA testing as the primary screening tool [4]. Current evidence indicates that HPV-based strategies offer superior preventive outcomes compared to traditional cytological approaches [5].

The economic burden of cancer care has become increasingly important in policy discussions of treatment value and resource allocation. In value-based healthcare, value is commonly understood as health outcomes in relation to the costs required to achieve them [6]. The evaluation of oncology care increasingly extends beyond clinical efficacy to include affordability and equity considerations. Financial toxicity is increasingly recognized as an important consequence of cancer care [7]. This burden may persist even in universal coverage settings; in Taiwan, severe financial hardship after cancer diagnosis has been associated with higher mortality even under the National Health Insurance (NHI) system [8]. Accurate stage-specific cost estimates are therefore important for economic evaluation and policy planning. Such estimates provide useful inputs for health economic modeling, budget planning, and evaluation of screening strategies [6,9,10]. From a value-based oncology perspective, stage-specific cost estimates are particularly important because they clarify how disease progression affects medical spending and how prevention or early detection may reduce the need for high-cost advanced-stage cancer care.

CC remains a persistent health challenge in Taiwan. National statistics show that 1384 new cases of CC were reported in 2022 and 620 related deaths were recorded in 2024, ranking it ninth for both incidence and mortality among women [11]. These figures reflect its ongoing public health burden. In response to this continuing burden, Taiwan expanded access to preventive screening in 2025. Under the current program, women aged 25–29 years are eligible for Pap smears every 3 years, whereas those aged 30 years and older are eligible for annual cytological screening. The expanded framework also includes age-specific HPV testing at 35, 45, and 65 years [11]. Primary prevention has also advanced, with the publicly funded HPV vaccination program extended to junior high school boys in 2025 [12]. Although Taiwan’s NHI system reduces patient cost-sharing through catastrophic illness certification, payer-side direct medical costs across different stages of cervical disease remain important to quantify [13]. Nevertheless, existing Taiwanese cost estimates were largely generated using earlier data and may not fully reflect recent changes in screening policy, HPV testing, vaccination expansion, treatment patterns, and healthcare utilization. In addition, updated evidence remains limited on direct medical costs across the full disease continuum from cervical intraepithelial neoplasia (CIN) to stage-specific invasive CC.

This evidence also has equity implications, as unequal access to vaccination, screening, diagnostic follow-up, and timely treatment may contribute to delayed diagnosis and a greater burden of advanced disease among underserved populations, even in a universal coverage system with substantial financial protection. This study used nationwide linked data to estimate healthcare utilization and expenditures associated with CIN and CC. By providing updated population-based cost estimates across CIN and stage I–IV invasive CC, the findings are intended to inform HPV vaccination, CC screening, and value-based prevention strategies that support more equitable allocation of healthcare resources in Taiwan.

## 2. Materials and Methods

### 2.1. Study Design and Perspective

This was a retrospective, population-based cost analysis using linked nationwide administrative and registry databases in Taiwan. The study was conducted from the payer perspective of Taiwan’s NHI program and focused on insurer-reimbursed direct medical costs for CIN and CC. The payer perspective was selected because Taiwan’s NHI is a single-payer system that finances most covered cervical disease-related medical services and is directly relevant to national budget planning, reimbursement policy, and prevention resource allocation. The analysis aimed to estimate stage-specific healthcare utilization and costs to inform value-based oncology and CC prevention policy. This study was approved by the Institutional Review Board of Fu Jen Catholic University (IRB No. C108121).

### 2.2. Data Sources

To ensure robust analysis, we utilized three nationwide databases available through the Health and Welfare Data Science Center, Ministry of Health and Welfare, Taiwan. These included the NHI Claims Database (2008–2017) for tracking healthcare utilization and reimbursed costs, the Taiwan Cancer Registry (TCR; both Annual Report and Long Form, 2008–2015) to pinpoint CC diagnoses and stages, and the National Cause of Death Registry (NCDR, 2008–2017) to verify survival outcomes. Together, these sources provide nearly 100% population coverage and are well-documented for their high validity and completeness. Database linkage was performed within the Health and Welfare Data Science Center, Ministry of Health and Welfare, using encrypted personal identifiers [14]. Researchers accessed only de-identified analytical files, and no directly identifiable personal information was available [14]. Linkage quality was supported by the use of nationally maintained administrative and registry databases with standardized coding systems [14,15,16,17,18].

### 2.3. Study Subjects

**Figure 1:**
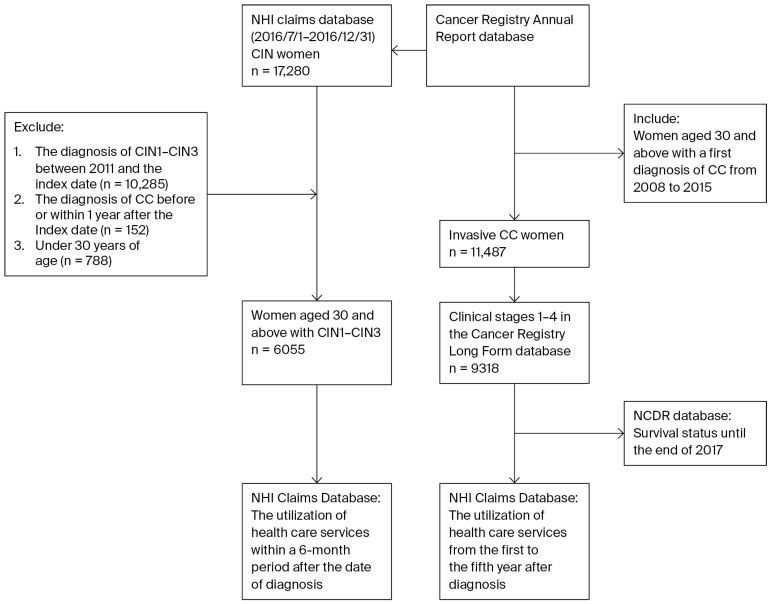
Flowchart illustrating the selection of participants with cervical intraepithelial neoplasia and cervical cancer.

Study subjects were divided into a CIN cohort and a CC cohort (Figure 1). For the CIN cohort, we identified women with CIN1, CIN2, or CIN3 diagnosed between 1 July and 31 December 2016 in the NHI Claims Database, based on outpatient or inpatient claims with ICD-10 codes N87.0, N87.1, or D06. The first eligible diagnosis was defined as the index date. This enrollment period was selected because the NHI Claims Database was available through 2017, thereby allowing a complete 6-month follow-up period after the index date for the assessment of CIN-related healthcare utilization and reimbursed costs. To identify incident CIN cases, we excluded women with any CIN diagnosis between 2011 and the index date, those with CC diagnosed before or within 1 year after the index date, and those younger than 30 years. Women younger than 30 years were excluded because Taiwan’s national organized cervical cancer screening program primarily targeted women aged 30 years and older during the study period. Women diagnosed with CC within 1 year after the CIN index date were excluded to reduce the likelihood of including prevalent, initially misclassified, or concurrently existing invasive cancer cases rather than incident CIN-related care. The final CIN cohort included 6055 women: 4484 with CIN1, 744 with CIN2, and 827 with CIN3.

For the CC cohort, we identified women with a first diagnosis of CC between 2008 and 2015 in the TCR Annual Report Database (ICD-O-3 code 180). This study period was defined by the availability of TCR data from 2008 to 2015, while linked NHI Claims Database and NCDR data available through 2017 allowed follow-up for cost estimation and survival ascertainment after diagnosis. Survival status was obtained from the NCDR, and clinical stage was identified from the TCR Long Form Database. For stage-specific analyses, we included women with invasive CC and complete stage information. The final analytic cohort comprised 9318 women with stage I–IV disease, including 3949 stage I, 2058 stage II, 1713 stage III, and 1598 stage IV cases.

### 2.4. Cost Estimation

Only direct medical costs reimbursed by the NHI were included. Outpatient and inpatient expenditures were defined according to reimbursed claim items recorded in the NHI Claims Database [14,15]. Outpatient costs included reimbursed ambulatory services related to cervical disease management, whereas inpatient costs included reimbursed hospitalization, procedures or surgery, medications, diagnostic tests, imaging, supplies, and other covered inpatient services. For the CIN cohort, costs were defined as reimbursed outpatient and inpatient expenditures incurred within 6 months after the index date for CIN-related care. Among the 6055 women with CIN, 5897 (97.4%) received treatment within 6 months after diagnosis. The 6-month follow-up window was selected to capture the initial diagnostic and treatment episode after CIN identification, following the costing approach used in previous Taiwanese research on cervical precancerous lesions, while ensuring complete follow-up for all included CIN patients within the available claims data [19].

For the CC cohort, annual direct medical costs were estimated from the first through the fifth year after diagnosis according to stage at diagnosis. Costs were derived from NHI claims for outpatient and inpatient care. No inflation adjustment was applied, and all costs are presented in nominal New Taiwan dollars (NTD), equivalent to US dollars at the 2017 exchange rate of NT$30.44 per US$1.00. Nominal costs were used to reflect actual reimbursed expenditures recorded by the NHI during the study period from the payer perspective.

### 2.5. Statistical Analysis

Healthcare utilization and costs are summarized using descriptive statistics. Given the right-skewed distribution of cost data, age-adjusted mean costs were estimated using generalized linear models with a gamma distribution and log-link function [20,21,22]. Age was included as the adjustment covariate because it is associated with cervical cancer stage, survival, and healthcare utilization.

For CC, cumulative annual costs were additionally estimated using the Kaplan–Meier sample average (KMSA) method to account for censoring [23,24]. Under the KMSA approach, interval-specific mean costs were weighted by the probability of surviving to the beginning of each monthly interval. Survival probabilities were derived from Kaplan–Meier curves based on linked mortality data from the NCDR, and patients who remained alive on 31 December 2017 were treated as censored observations. The KMSA method assumes that censoring is non-informative conditional on observed survival information and that the observed cost experience among patients at risk within each interval is representative of patients with incomplete follow-up. This approach was used to reduce bias caused by incomplete observation of costs among patients with different survival durations. Cumulative costs were estimated for each year from Year 1 through Year 5 after diagnosis. All statistical analyses were conducted using SAS software, version 9.4 (SAS Institute Inc., Cary, NC, USA).

## 3. Results

### 3.1. CIN-Related Healthcare Utilization and Costs

The healthcare utilization and reimbursed medical costs for outpatient and inpatient services among women with CIN and CC are summarized in Table 1 and Table 2. Because this study was designed as a descriptive population-based cost analysis, between-group differences are presented descriptively. Across CIN grades, no consistent gradient was observed in outpatient visits, cost per outpatient visit, length of hospitalization, cost per hospital day, or total cost per case. However, women with CIN1 had the lowest outpatient utilization, including fewer clinic visits, lower costs per visit, and lower total outpatient costs. In contrast, inpatient utilization was substantially higher among women with CIN3 than among those with CIN1 or CIN2, suggesting that high-grade precancerous lesions may require more intensive or procedure-based management in selected cases.

### 3.2. Stage-Specific Healthcare Utilization Among Patients with Invasive CC

Among women with CC, healthcare utilization was concentrated mainly in the first year after diagnosis across all disease stages. Outpatient utilization, measured by the proportion of patients with outpatient claims and the number of outpatient visits, was highest during the first year and declined thereafter across stages. Inpatient utilization generally increased with advancing stage during follow-up, although no clear stage-specific pattern was observed in the first year. The cost per inpatient admission was the highest among women with stage IV disease and was approximately 1.5- to 1.8-fold higher than that among women with earlier-stage disease. These findings indicate that advanced-stage disease was associated with greater inpatient resource intensity, particularly after the initial treatment year.

### 3.3. Stage-Specific Survival and Annual Medical Expenditures

Stage-specific survival and medical expenditures are detailed in Table 3. The age-adjusted estimates for first-year NHI-reimbursed costs reveal a sharp escalation in spending that tracks with disease progression, climbing from NT$256,095 for stage I to NT$474,724 for stage IV. The intermediate mean costs for stages II and III were identified as NT$385,235 and NT$448,422, respectively. The higher first-year costs in advanced-stage disease likely reflect greater treatment intensity, including more frequent outpatient care, hospitalization, procedures, systemic therapy, radiotherapy-related care, and supportive or palliative care needs. Regardless of the stage at diagnosis, medical spending was heavily concentrated in the first year. Adjusted annual mean expenditures were highest in the initial year after diagnosis across all stages and declined thereafter, with persistently higher expenditures in stage III and stage IV disease.

The five-year survival advantage diminished significantly as disease severity progressed. Rates fell from 85.3% in stage I to 68.0% and 54.5% for stages II and III, respectively, reaching a low of 19.5% for stage IV. Thus, more advanced stages were characterized by both higher early medical expenditures and poorer survival outcomes.

### 3.4. KMSA-Adjusted Cost Estimates

Regarding the economic modeling, the KMSA methodology consistently exceeded raw observed means when estimating the first-year medical burden across all diagnostic categories. These adjusted cost estimates were identified at NT$341,673 for stage I and rose to NT$450,749 for stage II, NT$515,751 for stage III, and NT$556,072 for stage IV. The higher KMSA-estimated costs in the first year may reflect the concentration of intensive diagnostic and treatment-related expenditures shortly after diagnosis. The gap between KMSA-estimated and observed mean costs narrowed over time. For stage IV disease, KMSA-estimated costs were lower than the observed mean costs from the second year onward. This pattern may reflect early mortality and survivorship-related selection in advanced diseases. In stage IV CC, patients who survived beyond the first year represented a smaller subgroup with observed ongoing care costs, whereas the KMSA approach weighted interval-specific costs by the probability of remaining alive at each interval.

## 4. Discussion

This population-based study, using nationwide medical insurance claims, cancer registry, and mortality data in Taiwan, provides a comprehensive assessment of healthcare utilization and direct medical costs across the spectrum of cervical disease, from CIN to CC [14,15,16,17,18]. Two main findings emerged. First, direct medical costs increased with disease severity, indicating a greater economic burden with more advanced disease [19,23]. Second, costs were concentrated in the period immediately following diagnosis, with the highest expenditures incurred during the first year and substantially lower costs thereafter [19,23]. Together, these findings indicate that disease progression is associated with a marked increase in financial burden, whereas the initial treatment period is the most resource-intensive phase of care.

Within the precancerous cohort, CIN1 accounted for 74% of aggregate medical resource use, primarily reflecting its higher case frequency rather than a greater per-patient burden. This pattern likely reflects Taiwan’s cervical screening program, identifying a substantial proportion of low-grade lesions before progression to higher-grade dysplasia [9,10,11,25]. Although management at this stage was common, the outpatient cost per visit increased with lesion severity, with median costs approximately doubling from CIN1 to CIN2. In contrast, inpatient utilization did not follow a strictly linear gradient across CIN grades, although CIN3 was associated with the highest per capita treatment cost. This pattern may be partly explained by clinical management differences across CIN grades. Current guidance indicates that CIN management may include ablative procedures and excisional procedures such as LEEP and conization [4]. Because higher-grade lesions, particularly CIN3, are more likely to require excisional management, and conization may be performed in outpatient, same-day, or short-stay inpatient settings, differences in procedure type and treatment setting may have contributed to the observed variation in inpatient utilization and costs [4,19].

Among patients with invasive cervical cancer, the economic burden was immediate and substantial. In the present study, the first 12 months after diagnosis accounted for 52% to 65% of the total treatment costs accrued over five years of follow-up. Relative to stage I disease, the first-year costs increased by factors of 1.5, 1.8, and 1.9 for stages II, III, and IV, respectively. Importantly, advanced-stage disease was associated with higher costs despite poorer survival, indicating that late-stage diagnosis may generate greater healthcare spending with less favorable outcomes. This finding reinforces the value of earlier detection and treatment. This front-loaded pattern is consistent with broader oncology literature, in which the initial phase of care is typically resource-intensive and advanced disease is associated with repeated hospitalization, systemic treatment, and palliative care needs [26,27,28]. For patients with CC, the concentration of first-year expenditures likely reflects intensive diagnostic evaluation and staging, initial surgery or chemoradiotherapy, hospitalization, and supportive or palliative care needs soon after diagnosis. Patient characteristics, including age and comorbidity burden, may further contribute to the variation in aggregate costs [27]. In addition, some patients with stage III or IV disease may receive a substantial proportion of treatment in ambulatory settings, particularly when chemoradiotherapy is delivered on an outpatient basis; therefore, inpatient utilization alone may not fully reflect treatment intensity. Methodologically, the use of the KMSA estimator strengthened the cost estimation by accounting for right-censored follow-up and improving estimation under incomplete observation [24]. These findings have important implications for healthcare planning, prevention policy, and cost containment. The concentration of first-year expenditures and the increase in costs with advancing stage highlight the importance of preventing delayed diagnosis and shifting detection and treatment toward earlier stages to reduce avoidable high-intensity treatment expenditures.

In many high-income settings, CC screening has shifted toward primary HPV testing [3,4,5]. In Taiwan, however, screening has historically relied on cytology-based approaches, and earlier studies suggested that uptake under this model remained suboptimal [9,10]. More recent official data indicate improved participation, with screening coverage among women aged 30–69 years reaching 70% under the national program [11,25]. At the same time, Taiwan has expanded its preventive policy framework by lowering the eligible age for Pap smear screening, introducing HPV testing for selected age groups, and broadening publicly funded HPV vaccination [11,12,25]. These developments suggest that screening uptake has improved in recent years, although further efforts are still needed to optimize participation and ensure appropriate follow-up after abnormal findings [11,25].

Taiwan has also experienced a marked long-term decline in CC burden. Official statistics indicate that the age-standardized incidence of CC decreased from 25.2 per 100,000 women in 1995 to 7.6 per 100,000 in 2022, whereas the age-standardized mortality rate decreased from 11 per 100,000 in 1995 to 2.5 per 100,000 in 2024 [29]. Because currently verifiable official data are reported as age-standardized incidence and mortality rates, temporal trends are more appropriately described using these indicators than by reference to a “7.7% deceleration” metric [29]. Despite this substantial progress, Taiwan has not yet reached the World Health Organization elimination threshold of fewer than 4 cases per 100,000 women, and further progress remains necessary [2,3,29].

The stage-specific cost estimates reported here are important because such parameters are key inputs for economic evaluations of HPV vaccination and CC screening strategies. Compared with earlier domestic estimates, the costs in the present analysis appear higher [19,23]. This difference may reflect temporal changes in diagnostic practice, treatment complexity, and medical pricing, although this interpretation should be made cautiously [19,23]. A direct comparison of medical costs across countries remains challenging because of differences in healthcare financing, costing methods, and included cost components. Such differences may reflect variation in payer coverage, reimbursement rules, treatment settings, hospitalization patterns, and whether studies include direct medical costs only or broader societal costs. Methodological choices, such as phase-specific versus stage-specific costing and whether censoring or survival time is considered, may also affect cross-study comparisons. Nevertheless, the overall pattern appears broadly consistent across settings. Population-based evidence from Canada has shown substantial initial-phase costs for CC care [30]. In the United States, newly diagnosed CC among commercially insured patients was associated with high first-year direct medical costs [31]. Studies from Mexico and France also reported considerable healthcare expenditure related to CC treatment and the management of squamous intraepithelial lesions [32,33]. Population-based data from Korea further support the substantial burden associated with CC at the national level [34]. Taken together, these findings indicate that CC imposes substantial clinical and economic burdens across health systems and underscore the importance of prevention and early detection [3,30,31,32,33,34]. These results support continued investment in HPV vaccination, HPV-based screening, and organized follow-up systems to reduce progression to advanced-stage disease, avoid high-intensity treatment expenditures, and improve survival and equity in cervical cancer control.

From the perspective of value-based oncology, healthcare value depends on the outcomes achieved relative to the resources consumed [6]. From a value-based perspective, prevention and early detection may improve healthcare value by shifting care toward earlier disease stages, where treatment is generally less resource-intensive and survival outcomes are more favorable, thereby reducing reliance on costly advanced-stage treatment. Although the present analysis was limited to NHI-reimbursed direct medical costs and did not capture patient-level financial toxicity, the findings remain relevant to discussions of financial protection [7,8,13]. Equity considerations are also relevant to these findings, as disparities in screening participation, follow-up care, and treatment access may contribute to delayed diagnosis and a greater advanced-stage disease burden even under universal health coverage. Strengthening HPV vaccination, HPV-based screening, and organized follow-up may therefore help reduce both avoidable treatment costs and healthcare inequities. Consistent with earlier Taiwanese studies, our results indicate that cervical disease imposes a substantial economic burden and that costs increase with advancing disease severity [19,23]. Given that expenditures in the present study were heavily concentrated in the first year after diagnosis, a five-year analytical horizon is likely to capture a substantial proportion, although not all, of disease-attributable direct medical costs [19,23].

Current guidance supports timely diagnostic evaluation and appropriate management after abnormal screening findings; however, this should not be interpreted as implying a universal requirement that all CIN lesions be definitively treated within six months of diagnosis [4,11]. Rather, the six-month analytical window used for precancerous disease in this study should be understood as a pragmatic costing horizon aligned with relatively near-term management in routine clinical practice, rather than as a formal treatment deadline [4,11]. This study has several strengths. It was based on robust, linked, nationwide datasets, thereby reducing the sampling bias commonly encountered in single-center analyses [14,15,16,17,18]. Several limitations should also be acknowledged. Case identification based on administrative coding remains subject to potential misclassification [14,15,16,17,18]. Information on treatment adherence, treatment completion, and detailed care pathways was not available; therefore, this study could not assess how adherence to recommended management influenced healthcare utilization or costs. In addition, because the analysis was conducted strictly from the NHI payer perspective, it did not include out-of-pocket expenditures, non-covered treatments, or broader societal costs such as productivity loss [13]. Therefore, the broader economic burden of cervical disease from patient or societal perspectives may be underestimated. Costs were also reported in nominal New Taiwan Dollars without inflation adjustment; therefore, comparisons across cohorts or time periods should be interpreted in light of secular changes in prices and clinical practice. For the CIN cohort, the 6-month follow-up window captured the initial diagnostic and treatment episode but may not fully reflect longer-term costs related to surveillance or delayed treatment. In addition, the adjusted analyses accounted for age only and did not incorporate other cost-related factors, such as comorbidity, treatment modality, socioeconomic status, and screening history; therefore, residual confounding may remain, and the estimates should be interpreted as age-adjusted rather than fully risk-adjusted. Although the KMSA method was used to account for incomplete follow-up in the CC cohort, no formal sensitivity analysis using alternative censoring-adjusted cost methods was conducted, limiting assessment of robustness under different censoring assumptions. Furthermore, confidence intervals for the age-adjusted and KMSA-estimated mean costs were not computed, which limits the evaluation of statistical variability around these point estimates. Future research should incorporate patient-level treatment patterns, adherence, out-of-pocket spending, indirect costs, and longer-term follow-up for CIN to provide a more comprehensive assessment of the clinical and economic burden of cervical disease. Despite these limitations, the present study provides a representative real-world financial baseline for cervical disease prevention and control in Taiwan. As health systems continue to expand HPV vaccination and move toward more sensitive molecular screening strategies, these cost estimates provide an empirical basis for the development of value-based and equitable prevention policies [3,4,12].

## 5. Conclusions

This nationwide study demonstrated that cervical disease imposes substantial direct medical costs on Taiwan’s healthcare system, with costs increasing with disease severity and being concentrated in the first year after diagnosis. These updated stage-specific estimates provide an empirical basis for HPV vaccination and CC screening policy and support the development of value-based CC prevention strategies in Taiwan.

## Figures and Tables

**Table 1:** Healthcare utilization in outpatients with cervical intraepithelial neoplasia or cervical cancer.

	Case Numbers	Outpatient						
		Usage	Number of Visits ^a^		Costs per Visit ^a^		Costs per Outpatient ^a^	
CIN								
CIN1	4484	98.6%	2	(1, 3)	1337.67	(498.50, 2767.00)	3171.0	(845.0, 5588.0)
CIN2	744	96.1%	3	(2, 5)	2897.50	(1194.00, 4115.20)	7441.0	(2853.0, 16,680.0)
CIN3	827	91.9%	3	(1, 5)	2487.00	(696.67, 3813.88)	8437.0	(1504.5, 16,675.5)
CC								
First year								
Stage 1	3852	97.7%	16	(9, 26)	2780.17	(1174.52, 9306.75)	36,256.5	(13,056.5, 256,198.0)
Stage 2	2042	98.5%	28	(20, 36)	9645.86	(7013.00, 12,820.63)	288,316.0	(196,016.0, 371,311.0)
Stage 3	1694	98.5%	31	(22, 40)	9365.36	(6828.89, 12,339.38)	307,526.5	(213,606.0, 385,785.0)
Stage 4	1582	93.0%	27	(14, 39)	8502.74	(5107.50, 12,210.00)	249,793.0	(87,119.0, 385,517.0)
Second year								
Stage 1	3758	91.0%	8	(5, 13)	1223.57	(776.26, 1973.30)	10,225.0	(4768.0, 19,938.0)
Stage 2	1885	92.4%	11	(7, 17)	1526.10	(951.73, 2491.76)	16,356.0	(8019.0, 31,406.0)
Stage 3	1450	93.3%	13	(8, 21)	1738.62	(1093.60, 3051.00)	21,579.0	(11,705.0, 49,554.0)
Stage 4	875	89.9%	14	(8, 24)	2298.73	(1392.95, 4233.85)	30,802.0	(13,696.0, 73,337.0)
Third year								
Stage 1	3600	85.4%	6	(4, 11)	1175.25	(756.33, 1940.00)	8005.0	(3409.0, 16,077.0)
Stage 2	1672	87.3%	9	(5, 14)	1416.73	(848.00, 2374.00)	12,165.0	(5302.0, 23,409.0)
Stage 3	1213	89.9%	10	(5, 17)	1570.00	(966.45, 2666.90)	15,237.0	(6999.0, 32,697.0)
Stage 4	561	88.2%	12	(6, 20)	2124.50	(1221.60, 3577.93)	23,993.0	(10,634.0, 54,549.0)
Fourth year								
Stage 1	3154	81.0%	5	(3, 10)	1104.93	(726.82, 1860.88)	6209.0	(2848.0, 13,972.0)
Stage 2	1359	83.7%	7	(4, 13)	1283.00	(792.00, 2242.50)	10,067.5	(4249.0, 19,508.0)
Stage 3	907	84.2%	8	(5, 15)	1481.65	(920.65, 2481.59)	13,439.0	(5766.5, 24,563.0)
Stage 4	358	83.8%	10	(5, 20)	1814.94	(1195.83, 2970.13)	17,733.5	(8931.0, 47,546.0)
Fifth year								
Stage 1	2713	76.4%	5	(2, 8)	1083.00	(705.63, 1866.00)	5099.0	(2363.0, 12,733.0)
Stage 2	1119	78.8%	6	(4, 11)	1237.42	(782.67, 2172.42)	9006.5	(3580.0, 17,923.0)
Stage 3	726	80.7%	6.5	(4, 13)	1408.08	(811.00, 2368.67)	10,052.5	(3880.0, 19,923.0)
Stage 4	250	84.4%	9	(4, 18)	1552.52	(941.50, 2515.33)	14,045.0	(5474.0, 33,695.0)

^a^: Median (interquartile range). CIN, cervical intraepithelial neoplasia; CC, cervical cancer.

**Table 2:** Healthcare utilization in inpatients with cervical intraepithelial neoplasia or cervical cancer.

	Case Numbers	Inpatient						
		Usage	Hospital Days ^a^		Costs per Hospital Day ^a^		Costs per Inpatient ^a^	
CIN								
CIN1	4484	0.6%	4	(3, 5)	15,840.30	(12,348.10, 18,840.75)	63,622.5	(48,577.0, 75,363.0)
CIN2	744	5.5%	1	(1, 4)	15,165.00	(11,664.33, 18,113.00)	19,524.0	(16,681.0, 60,409.0)
CIN3	827	18.9%	4	(2, 5)	15,819.60	(10,120.00, 20,107.38)	68,442.0	(42,550.5, 78,109.5)
CC								
First year								
Stage 1	3852	86.1%	11	(7, 18)	10,096.03	(7992.56, 12,598.85)	121,607.0	(90,684.5, 159,711.0)
Stage 2	2042	74.3%	11	(6, 21)	8915.58	(6660.75, 12,004.50)	111,502.0	(51,305.0, 187,370.0)
Stage 3	1694	81.4%	13	(7, 29)	8711.38	(6470.46, 11,469.10)	127,085.0	(58,108.0, 246,288.0)
Stage 4	1582	85.8%	25	(11, 47)	7690.43	(5922.94, 10,205.92)	195,987.5	(86,123.0, 369,157.0)
Second year								
Stage 1	3758	10.0%	12	(5, 31)	6803.54	(4689.43, 10,697.75)	97,782.0	(29,780.0, 240,668.0)
Stage 2	1885	17.7%	16	(6, 34)	6606.07	(4416.80, 9404.82)	94,423.0	(41,125.0, 245,442.0)
Stage 3	1450	28.0%	19	(9, 41)	6924.34	(4575.94, 10,088.38)	140,355.0	(57,528.0, 283,884.0)
Stage 4	875	46.7%	22	(8, 41)	6128.00	(4409.00, 9097.20)	138,975.0	(55,377.0, 260,796.0)
Third year								
Stage 1	3600	7.6%	15.5	(6, 34)	7061.72	(4671.77, 10,178.80)	122,921.5	(35,942.5, 254,482.5)
Stage 2	1672	12.4%	17	(7, 42)	6734.82	(4309.67, 10,562.42)	104,364.0	(47,931.0, 299,195.0)
Stage 3	1213	17.9%	16	(6, 40)	7250.00	(5256.42, 11,047.43)	118,988.0	(50,150.0, 291,181.0)
Stage 4	561	31.0%	17	(7, 37)	6318.01	(4458.20, 8717.29)	109,492.0	(48,305.0, 264,181.0)
Fourth year								
Stage 1	3154	5.8%	15	(5, 37)	6810.02	(4633.03, 10,703.94)	123,983.0	(33,653.0, 305,057.0)
Stage 2	1359	8.8%	11	(5, 25.5)	5652.83	(4179.25, 9992.34)	74,768.5	(33,827.5, 173,812.5)
Stage 3	907	13.9%	15	(6, 38)	7024.95	(3991.77, 10,391.56)	104,176.5	(35,853.0, 250,935.0)
Stage 4	358	24.6%	15	(8, 31)	6151.25	(4039.80, 9119.57)	104,408.0	(36,266.5, 210,250.5)
Fifth year								
Stage 1	2713	3.4%	16.5	(5.5, 33.5)	6774.92	(4783.84, 11,171.23)	103,844.0	(49,223.0, 266,442.5)
Stage 2	1119	6.8%	17.5	(6.5, 35.5)	6959.25	(4496.50, 10,739.99)	116,828.0	(39,535.5, 237,269.5)
Stage 3	726	8.1%	14	(5, 31)	7815.00	(5015.70, 13,809.64)	103,848.0	(44,776.0, 243,071.0)
Stage 4	250	16.8%	9	(6, 22)	5961.29	(4525.00, 11,997.77)	70,530.5	(38,534.0, 141,246.0)

^a^: Median (interquartile range). CIN, cervical intraepithelial neoplasia; CC, cervical cancer.

**Table 3:** Total medical expenditure for 5 years in cervical cancer patients (NT$).

Stage	Period	Observed Mean Cost		Survival Rate (%)	KMSA ^b^ Estimation Cost
		Unadjusted	Adjusted ^a^		
I	1 Year	255,801.54	256,094.70	97.7%	341,673.19
	2 Year	45,997.11	46,020.97	93.7%	94,415.75
	3 Year	38,587.26	38,708.36	90.3%	88,630.43
	4 Year	34,132.64	34,226.28	87.2%	86,346.30
	5 Year	25,469.39	25,540.00	85.3%	69,149.07
II	1 Year	385,398.00	385,234.57	93.7%	450,748.87
	2 Year	72,865.65	72,621.15	82.3%	118,830.18
	3 Year	57,746.85	57,580.96	75.6%	96,141.42
	4 Year	36,998.08	36,898.01	70.8%	66,993.10
	5 Year	39,196.70	38,974.05	68.0%	69,234.17
III	1 Year	448,430.03	448,422.28	87.5%	515,750.69
	2 Year	114,225.14	113,484.58	72.8%	155,954.23
	3 Year	78,378.76	77,863.31	63.5%	106,917.25
	4 Year	63,703.97	62,416.72	58.1%	84,830.46
	5 Year	47,111.29	46,536.14	54.5%	66,895.51
IV	1 Year	475,237.09	474,723.86	58.3%	556,071.82
	2 Year	172,700.30	172,789.59	36.5%	13,3263.44
	3 Year	116,226.70	116,141.87	27.3%	68,001.93
	4 Year	87,183.25	87,098.58	22.2%	42,784.63
	5 Year	64,752.30	64,551.67	19.5%	28,961.38

^a^: Adjusted for age by the generalized linear model with log-link function and gamma distribution. ^b^: KMSA, Kaplan–Meier sample average.

## Data Availability

The datasets generated and/or analyzed during the current study are not publicly available in accordance with the policy of the Health and Welfare Data Science Center, Ministry of Health and Welfare, Taiwan.
